# Molecular Mechanisms of Parthanatos and Its Role in Diverse Diseases

**DOI:** 10.3390/ijms23137292

**Published:** 2022-06-30

**Authors:** Ping Huang, Guangwei Chen, Weifeng Jin, Kunjun Mao, Haitong Wan, Yu He

**Affiliations:** 1 School of Pharmaceutical Sciences, Zhejiang Chinese Medical University, Hangzhou 310053, China; 202011014011078@zcmu.edu.cn (P.H.); 201811114011456@zcmu.edu.cn (G.C.); 20091019@zcmu.edu.cn (W.J.); 20211106@zcmu.edu.cn (K.M.); 2 School of Life Sciences, Zhejiang Chinese Medical University, Hangzhou 310053, China

**Keywords:** parthanatos, hallmarks, molecular mechanisms, related diseases

## Abstract

Differential evolution of apoptosis, programmed necrosis, and autophagy, parthanatos is a form of cell death mediated by poly(ADP-ribose) polymerase 1 (PARP1), which is caused by DNA damage. PARP1 hyper-activation stimulates apoptosis-inducing factor (AIF) nucleus translocation, and accelerates nicotinamide adenine dinucleotide (NAD^+^) and adenosine triphosphate (ATP) depletion, leading to DNA fragmentation. The mechanisms of parthanatos mainly include DNA damage, PARP1 hyper-activation, PAR accumulation, NAD^+^ and ATP depletion, and AIF nucleus translocation. Now, it is reported that parthanatos widely exists in different diseases (tumors, retinal diseases, neurological diseases, diabetes, renal diseases, cardiovascular diseases, ischemia-reperfusion injury...). Excessive or defective parthanatos contributes to pathological cell damage; therefore, parthanatos is critical in the therapy and prevention of many diseases. In this work, the hallmarks and molecular mechanisms of parthanatos and its related disorders are summarized. The questions raised by the recent findings are also presented. Further understanding of parthanatos will provide a new treatment option for associated conditions.

## 1. Introduction

Parthanatos, a kind of new programmed death mode, has been put forward by Professors Ted and Valina Dawson to indicate a caspase-independent cell death subroutine that critically relies on the hyper-activation of poly(ADP-ribose) polymerase 1 (PARP1) [1,2]. Traumatic injury, excitotoxicity, ischemia, and many neurodegenerative disorders will cause toxic stimuli accumulation, which leads to DNA damage, PARP1 hyper-activation, PAR accumulation, and mitochondrial apoptosis-inducing factor (AIF) nucleus translocation. Ultimately, large-scale DNA fragmentation and later caspase activation lead to mitochondria dysfunction, failure to generate NAD^+^/ATP, and end up with cell collapse.

**Figure 1:**
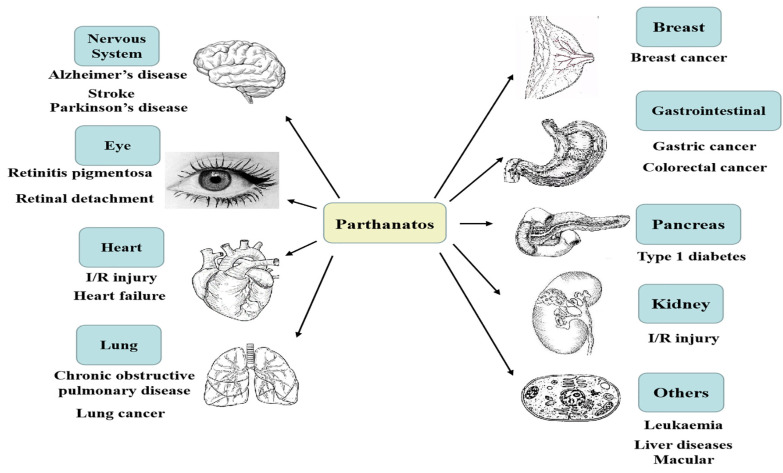
Parthanatos is associated with multiple system diseases; for example, nervous system diseases, heart diseases, retinal diseases, gastrointestinal diseases, lung diseases, kidney diseases, pancreatic diseases, breast diseases, and so on.

There are many differences between some common forms of cell death and parthanatos in morphological features, biochemical features, regulatory pathways, key genetic inhibitors, or inhibition by protein over-expression (Table 1). Parthanatos is characterized by PARP1 hyper-activation, PAR polymers accumulation, mitochondria depolarization, and AIF nucleus translocation [3,4,5]. It is reported that parthanatos is involved in many common important diseases, such as Parkinson’s disease, stroke, cancer, heart attack, retinal disease, and diabetes [6,7,8,9,10], and can be applied to the clinical treatment of related diseases (Figure 1).

In this work, we review the hallmarks and molecular mechanisms of parthanatos and its related diseases to further learn about its pathogenesis and put forward new treatments for related conditions.

## 2. Hallmarks of Parthanatos

### 2.1. Morphological Features

Cells undergoing parthanatos usually show necrosis-like and apoptosis-like morphological changes [2]. These features include the loss of cell membrane integrity, cellular propidium iodide (PI) positive staining, and DNA fragmentations (15 kb to 50 kb) [1,11]. At the ultra-structural level, parthanatic cells usually exhibit mitochondrial abnormalities, such as the dissipation of inner transmembrane potential, nuclear shrinkage, and chromatin condensation [1].

### 2.2. Biochemical Features

#### 2.2.1. DNA Injury

DNA injury signaling is essential in maintaining genome integrity and cell fate. The main reasons for DNA injury are roughly divided into environmental effects (exogenous damage) and spontaneous injury (endogenous injury). Environmental factors often cover ultraviolet radiation (UVR), ionizing radiation (IR), alkylating agents, and metabolically activated compounds. Mistakes in DNA replication, base tautomerism, base deamination, and loss belong to spontaneous DNA damage. Sometimes chronic UVR is helpful to DNA repair response; most of the time, it results in unrepairable DNA damage [12,13,14]. One publication emphasizes mitochondrial changes and DNA damage mediated by UVR in HaCaT cells, and the significance of PARP in these alterations [15].

IR (α-, β-, γ-, X-ray), a valid and widely used measurement for cancer treatment, is able to control tumors affecting DNA damage response and repair (DRR) processes, which determine the fate of the tumor cells. IR-induced DNA double-strand breaks (DSBs) are the most lethal form of damage [16].

Regarding alkylating agents, the primary mode of their action causes cytotoxic DNA damage. To resist alkylation-induced cell death or mutation, direct DNA damage reversal, base excision repair (BER), and mismatch repair (MMR) come into work. It is essential for a favorable response of the organism to alkylating agents to keep an appropriate balance of activity both within and between these pathways [17]. Widespread as an environmental mutagen, N-methyl-N′-nitro-N-nitrosoguanidine (MNNG) is a commonly used DNA-alkylating agent that potently initiates parthanatic cell death in cell-death research [18].

ROS play a multifaceted and pleomorphic role in DNA damage response (DDR), such as mediating genotoxin-induced damage, DNA damage by oncogenic replication stress, sensing of DSBs, signal transduction within DDR, cell cycle progression, apoptosis, and DNA repair. It is necessary to distinguish the role between oxidative stress and ROS in DDR [19,20,21].

PARP1 is crucial in maintaining genomic stability by facilitating DNA repair to ensure cell survival. It can regulate BER, single-stranded break (SSB), and DSB repair pathways. Meanwhile, PARP1 mediates parthanatos in response to severe DNA damage [22].

#### 2.2.2. NAD^+^ Depletion

Cellular energy depletion is caused by PARP1 over-activation through NAD^+^ consumption [23,24]. NAD^+^ is a co-factor in cellular metabolism, which is needed for generating ATP. Besides, NAD^+^ resynthesis calls for about 2–4 molecules of ATP [25]. Studies suggest that ATP decreases with the consumption of NAD^+^, along with a decrease in cellular energy, resulting in cell death [26,27]. NAD^+^ and energy are preserved when PARP1 inhibitors and the model of PARP1 deficiency are used [28,29]. However, the decrease in NAD^+^ is not always associated with ATP decline in cerebral ischemia-reperfusion injury (CI/RI) models. A study points out that PARP1 KO mice reduce the infarct size in the CI/RI group, compared with the normal group, but the energy status does not change [30].

#### 2.2.3. Poly(ADP-Ribose) (PAR) Accumulation

The synthesis of PAR polymers depends on certain enzymes called PARPs [31]. PARP1, a member of PARPs, is the most studied and makes the greatest contribution to synthesizing PAR polymers [32]. When there is a toxic stimulus, DNA is damaged, PARP1 becomes hyper-activated and generates superfluous PAR polymer, followed by AIF nucleus translocation [33]. PAR polymers activate a signal to modulate the downstream transcriptional process and the mechanism of DNA repair. PAR interacts with NONO, a novel PAR-binding protein, the binding modulates the physiological functions of protein [34]. Subsequently, PAR has been studied to be directly toxic to neurons. AIF and PAR polymer-binding protein [35,36], a physical interaction between PAR and AIF, induces AIF release from the mitochondria and leads to the occurrence of parthanatos [37,38].

### 2.3. Genetic Features

A few genes/proteins are considered biomarkers of parthanatos; for example, PARP1 is a therapeutic target in the stroke, trauma, I/R injury, and diabetes model [23]. Cell parthanatos is suppressed in fibroblasts that are isolated from PARP1 KO mice [39]. AIF is identified as a candidate, PAR-binding protein [36,37]. Genetic ablation of NAMPT or FK866 treatment sensitizes lymphocytes to MNNG-induced parthanatos, while over-expression of a catalytically active recombinant NAMPT protects NIH-3T3 cells from the toxicity of the same DNA alkylating agent [40]. The result of AIF KO shows AIF as a cell death effector in PARP1 toxicity and parthanatos [41]. KO PARG dysfunction sensitizes various cancer cells to chemotherapeutic agents and radiation [42,43]. Human osteosarcoma U2OS is exposed to hydrogen peroxide (H_2_O_2_) and is compared with the ADP-ribosylation (ADPr) pattern of control, ADP-ribosyl hydrolase 3 (ARH3) KO, HPF1 KO, and PARP1 KO cells. The data indicate that global ADPr in response to DNA damage requires ARH3, HPF1, and PARP1 [44].

### 2.4. Immune Features

The activation of PARP1 promotes the transcription of pro-inflammatory genes and down-regulates multiple pathways of inflammation and tissue injury [45]. The cells, which infect oligodendrocytes in human natural killer (NK) cells, are killed by virus-induced PARP1 and AIF translocation, rather than the immune system [46]. For instance, the novel mechanism of immune escape in acute myeloid leukemia (AML) is uncovered: mature malignant cells in monocytic forms of leukemia have the capacity to produce ROS via NADPH oxidase and thus trigger parthanatos in adjacent antileukemic lymphocytes [47]. In addition, the lymphocyte function is impaired because those malignant ROS-producing myeloid cells induce parthanatos in NK cells [47,48,49]. NAD^+^ metabolism makes a contribution to inflammation and immune responses [50]. Human cytomegalovirus (HCMV) also restrains local immune responses through ROS-induced parthanatos [14]. PARG is vital to tumor growth and metastasis of colon carcinoma and may be applied as a new target for treating colon carcinoma [51].

## 3. Molecular Mechanisms of Parthanatos

**Figure 2:**
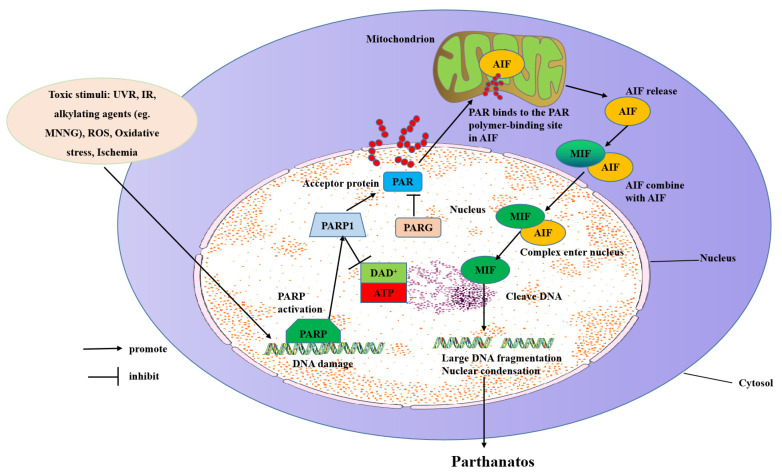
Molecular mechanisms of parthanatos. Toxic stimuli such as ROS, ischemia, alkylating agents (e.g., MNNG), IR, and UVR activate PARP-1 directly or indirectly through activation of NOS, which makes NO to induce ROS and the subsequent DNA damage. PARP-1 overactivation produces free PAR by PARG-mediated hydrolyzation, which serves as a death signal from the nucleus to mitochondria, inducing the release of AIF. AIF then translocates with MIF to the nucleus where it induces extensive fragmentation of DNA. This form of cell death is called “parthanatos”.

The molecular mechanisms of parthanatos include DNA damage, PARP1 hyper-activation, NAD^+^ and ATP depletion, and AIF translocation from the mitochondrial to the nucleus (Figure 2).

### 3.1. Inducing Parthanatos by Injuring DNA

DNA damage is caused by toxic stimuli, such as H_2_O_2_ or hydroxyl radical, nitrosative stress from NO or peroxynitrite (ONOO^−^), inflammation, ischemia or I/R, hypoxia, hypoglycemia, and DNA-alkylating agents [25]. There are many DNA repair pathways, including BER, nucleotide excision repair (NER), mismatch repair (MMR), homologous recombination (HR), and non-homologous end-joining (NHEJ). These pathways help to repair DNA damage throughout different stages of the cell cycle [52].

PARP1 is a kind of enzyme that takes part in the process of DNA repair. It may not catalyze the process of DNA repair directly; however, the process of activating multiple DNA repair enzymes such as DNA topoisomerase, DNA helicase, and DNA ligase needs PARP1′s participation [45]. PARP1 composes a DNA base-excision repair system through testing the crack and fracture of DNA strands and promoting its repair via PAR polymer synthesis. When DNA is mildly damaged, PARP1 is hundreds of times more active, and the enzyme uses NAD^+^ to synthesize PAR polymer by consuming ATP. However, when there is serious DNA damage, PARP1 is over-activated and PAR polymers are generated and accumulated, which leads to phosphatidylserine externalization, mitochondrial membrane potential (MMP) dissipation, AIF nucleus translocation, massive DNA fragmentation, and chromatin condensation, and then parthanatos occurrence [1,2]. PAR also participates in DNA replication and repair; the cell, which is inhibited by PARG, shows an increased sensitivity to DNA-damaging agents [10]. PARG is conducive to DSB and SSB repair, recovery from prolonged replication stress, and unusual replication structures in the S phase [53].

### 3.2. Inducing Parthanatos by Hyper-Activating PARP1

PARP1, a highly expressed 116 kD eukaryotic nuclear protein, includes an N-terminal, a self-modifying domain, and a C-terminal catalytic domain. In response to DNA damage, histone PARylation factor 1 (HPF1), a required co-factor in PARP1/PARP2-mediated ADP-ribosylation of serine, directly binds to the catalytic domain of PARP1 via its C-terminal domain. The active site, which is formed by HPF1 and PARP1, activates the PARP1 enzyme [54]. PARP1 combines with DNA primarily by the second zinc-finger domain [45,55,56], it catalyzes NAD^+^ and synthesizes PAR polymers adhered to various nuclear proteins, which significantly influences their function [12].

PARP1 is vital in repairing DNA, leading to stability and transcription of genomics with normal physiological conditions. When DNA damage is slight, PARP1 is more reactive, resulting in ADP-ribosylation of PARP1 and its substrates, which then helps to recruit DNA repair effector proteins to repair DNA [57]. Nevertheless, under immoderate genotoxic stress, PARP1 is hyper-activated and generates excessive PAR and stimulates AIF nucleus translocation, causing a sharp drop in NAD^+^ storage after DNA breaks, which results in parthanatos. PARP1 is activated by DNA damage, which is caused by external stimuli. Furthermore, PARP1 is stimulated by the calcium (Ca^2+^) signaling pathway [9].

PARP1 hyper-activation is the first and critical step in the parthanatic cascade. The inhibition or deficiency of PARP1 is protective in models of the cell and rat/mouse injury paradigms, including retinal degeneration diseases, stroke, diabetes, I/R injury, neurodegenerative disease, and heart failure [58]. PARP1 KO mouse models are hypersensitive to alkylating agents and IR, which is related to defective DNA repair [59]. On the contrary, PARP1 KO mouse models are protected from LPS-induced shock, ischemic injury, and 1-methyl-4-phenyl-1,2,3,6-tetrahydropyridine (MPTP) excitotoxicity. Therefore, these genetic models emphasize a double function of PARP1 in the cell depending on the stimulation: promotion of DNA repair or mediation of pathological cascades of inflammation/excitotoxicity [9]. These studies contribute to understanding the role of PARP1 in pathological contexts and related diseases. In preclinical zebrafish and human organotypic 3D skin models of psoriasis, PARP1 hyper-activation reacts to ROS-induced DNA damage: it is fueled by nicotinamide phosphoribosyltransferase-derived NAD^+^ and mediates inflammation through parthanatos [60]. In short, as a cell fate determinant, PARP1 has a significant influence in promoting DNA repair or restraining parthanatos. Now, many researchers have presented a number of potential treatments, of which the PARP1 enzyme has been regarded as a potential target intended to deal with related diseases. Targeting various diseases with chemical inhibitors of PARP1 provides therapeutic outcomes by reducing PARP1-mediated neuronal death. Many PARP1 inhibitors (oxaliplatin, PJ-34, 3-aminobenzamide, olaparib) have been studied in stroke, Parkinson’s disease (PD), Alzheimer’s disease (AD), Huntington’s disease (HD), and amyotrophic lateral sclerosis (ALS), but have not been clinically evaluated [61].

### 3.3. Inducing Parthanatos by Binding of PAR

Nuclear DNA damage stimulation triggers the synthesis of poly ribose (ADP-ribose), next to the distribution of the PAR polymer to the cytoplasm and mitochondria, and then the release of AIF [13]. PAR inhibits the glycolysis process. ARH3 hydrolyzes protein-free PAR in the nucleus and cytoplasm, inhibits PAR transfer to the cytoplasm, and thus prevents parthanatos [62]. ARH3 has been shown to catalyze PAR-degradation in vitro [63,64]. PARG, the most well-characterized enzyme in humans for PAR hydrolysis, utilizes a macrodomain fold to bind ADP-ribosylation and specifically cleaves the ribose-ribose bonds between the subunits of the PAR chains. PARG needs the cooperation of PARP to repair DNA. Once the strand of PARP and DNA breaks, an enzyme is activated, causing PARP to shuttle and the chromatin to open. PARG enters the nucleus and moves to the PARP substrate to repair the broken DNA strand. With the increase in PARG and the decrease in PAR, chromatin recovers its original structure. PARG has been shown to play a vital role in various diseases [65].

### 3.4. Inducing Parthanatos by Depleting ATP and NAD^+^

NAD^+^ is a factor of ATP generation and its resynthesis needs many molecules of ATP, which its consumption results in ATP depletion and cellular energy downturn, which leads to parthanatos [9,28]. The inhibition of PARP1 by pharmacological inhibitors or genetic deletion could recover NAD^+^ levels [28]. Neurons that are stimulated with toxic show protection and accompanying energy conservation with PARP inhibitors. Besides, PARP1 KO mice of transient cerebral ischemia-induced damage present preserved NAD^+^ levels [29,66], supporting the suicide hypothesis of PARP1 activation causing a block in glycolysis. Besides, adding NAD^+^ to cells or over-expression of NAD^+^ avoids PARP1-dependent parthanatos, which suggests that the decreased NAD^+^ relevant to PARP1 hyper-activation causes cell demise [28].

A recent finding suggests that the mitochondrial pool of NAD^+^ is associated with parthanatos because that parthanatos is saved by the preservation of NAD^+^ in the mitochondria by adding NAD^+^ biosynthetic enzyme Nampt or MPT inhibitor cyclosporine-A (CsA), and replenishing NAD^+^ [67,68]. The decrease in NAD^+^ after PARP1 hyper-activation reflects the level of NAD^+^ in the whole cell. Therefore, the conclusions of these studies need to be re-examined as the research shows that mitochondrial NAD^+^ is still at physiological levels after genotoxic stress, and even the nucleus and cytoplasmic NAD^+^ are depleted [68]. There are no reports yet to confirm that PARP1-dependent parthanatos entirely depends on mitochondrial NAD^+^ depletion [69]. Cyclosporine A may reduce parthanatos because it preserves mitochondrial NAD^+^ and inhibits mitochondrial permeability transitions, which are themselves an essential participant in parthanatos [67].

Researchers have questioned whether PARP1 hyper-activation was killed mainly by NAD^+^ depletion. NAD^+^ descent is not always associated with ATP decline in the I/R model [70]. Moreover, the energy status does not change as the infarct size is reduced in PARP1 KO mice after MCAO compared with normal mice [71]. In addition, Bax and calpain KO cells are protected from MNNG to the same degree as wild-type controls treated with the PARP1 inhibitor DPQ [72]. Although the NAD^+^ level is decreased in Bax and calpain KO cells, it is not in DPQ-treated cells. These results imply that PARP1 activation, which is caused by NAD^+^ depletion, is insufficient to account for parthanatos. NAD^+^ is expended by ADP-ribose transferases (ARTs) and many PARPs to generate an ADP-ribose protein modification and form PARP1 [73]. cADP-ribose synthases generate and hydrolyze the Ca^2+^-mobilizing second-messenger cADP-ribose from NAD^+^ [25,69,74]. There are two ways to promote the increase in NAD^+^ levels: stimulating the synthesis of NAD^+^ or inhibiting its excessive consumption [25,75,76].

### 3.5. Inducing Parthanatos by Releasing and Translocating AIF from Mitochondrial to the Nucleus

Mitochondria is a crucial element in the regulation of parthanatos. Its inter-membrane gap contains a number of proteins released through the outer membrane for taking part in parthanatos degradation. Mitochondria maintain the physiological integrity of cells by energy generation. However, once a cell is stimulated, the mitochondria become permeabilized, and AIF is released to irritate parthanatos. The change of outer membrane permeabilization (OMP) and permeability transition pore (PTP) will lead to the release of pro-apoptotic factors [77]. Bax, one of the pro-apoptotic members, is downstream of PARP1 and induces OMP needed for AIF release. Besides OMP, AIF cleavage by calpains requires AIF to be entirely released from the mitochondria [2].

According to the function, AIF is divided into three parts: an N-terminal part (binds to FAD), a central portion (which binds to NAD or NADH, which have oxidoreductase ability), and a C-terminal part (which is related to parthanatos) [26]. The harlequin (Hq) mouse is a helpful model to research AIF-mediated parthanatos [78]. It is noteworthy that AIF translocation is a vital step for parthanatos [33,79], so the understanding of this event is important in translating parthanatos research into therapy. In the nucleus, AIF-binding DNA is considered necessary even for parthanatos precipitation [80]. AIF, a parthanatos effector, takes part in the process of generating mitochondrial energy. It is usually restricted to mitochondria but translocates to the nucleus when parthanatos happens. [81,82]. AIF translocation, a marker of parthanatos, usually occurs in conjunction with the detection of specific mitochondrial markers. AIF contains a PAR-binding motif, facilitating the direct association between PARP1 and AIF [36], and takes part in PARP1 toxicity [82]. When PARP1 is hyper-activated, excessive PAR escapes from the nucleus and binds to specific cytosolic or mitochondrial proteins [35,38]. PAR combines with these proteins and eventually causes AIF to be released from the mitochondria.

AIF’s structure is markedly regulated by binding NAD(P)H and forming a stable, long-lived charge-transfer complex (CTC). In the process of PARP1-initiated parthanatos, once the NAD^+^ level is critically depleted, structural modulation of NADH regulates AIF release from mitochondria [83,84]. Excessive PAR triggers the mitochondrial release of AIF, which combines with macrophage MIF and carries MIF into the nucleus where the combination cleaves genomic DNA into large fragments. Chromatinolysis could be inhibited by depleting MIF and/or disrupting the AIF-MIF interaction then preventing MIF nuclease activity. The prevention of MIF nuclease activity should be a fascinating target for diseases.

## 4. Parthanatos and Related Diseases

Parthanatos is found in many diseases. Excessive or defective parthanatos will cause pathological cell changes. In this work, we sum up the relationship between parthanatos and cancer, retinal disease, diabetes, renal disease, heart failure, myocardial infarction, leukemia, lung injury, smoke-related lung diseases, stroke, ischemic tissue injury, brain trauma, and neurodegenerative diseases, and some representative agents of the parthanatos-associated components of targeted therapy (Table 2).

### 4.1. Parthanatos and Tumors

#### 4.1.1. Breast Cancer

The high expression of PARP1 is linked to the scarcity of apoptotic bodies necrosis, which is the cytomorphological feature of parthanatos [129]. PARP1 inhibitors have been well used in clinical practice. For example, talazoparib has a better effect than standard chemotherapy on progression-free survival in patients with advanced BC [85]. BZL101 [86], an aqueous extract from *Scutellaria barbata*, inhibits BC by promoting AIF translocation and has been verified to be feasible and safe for advanced BC patients in a phase I clinical trial. Ganetespib [87], GA/17AAG [88], and lapatinib [89] show evidence of activity in metastatic HER2-positive and triple-negative BC in a trial through stopping MIF stabilization.

#### 4.1.2. Colon Cancer

The E26 transformation-specific (ETS) factor inhibitor represents a promising therapeutic option for p53-deleted colon cancer. ETS1 over-expression induced by MAPK hyper-activation is thought to result in p53 loss/deregulation, PARP1 over-expression, and consequent YK-4-279-mediated parthanatos induction [130]. As the first PARP1 inhibitor, AG14361 has the pharmacologic properties of high potency, specificity, and stability, and plays a radiation sensitizer to reinforce radiation-induced parthanatos by inhibiting DNA repair [90]. HMA (5-(N,N-hexamethylene amiloride), a sodium-hydrogen antiporter inhibitor, promotes the induction of the parthanatos pathway (PAR accumulation and the translocation of the mitochondrial protein AIF to the nucleus) in SW613-B3 colon carcinoma cells [91].

#### 4.1.3. Ovarian Cancer

In studies of ovarian cancer, three PARP1 inhibitors, olaparib [92], niraparib [93], and rucaparib [94] exploit synthetic lethality in platinum-sensitive, relapsed serous ovarian cancer. PDD00017273, a quinazolinedione, suppresses PARG, stabilizes cellular PAR chains, and is devoid of activity against PARP1 and the ARH3 glycohydrolase. It uses and intensifies DNA replication deficiencies and then leads to ascended DNA damage in ovarian cancer cells [95,96]. COH34 [131], a PARG inhibitor, extends the arylation of DNA damage and captures DNA repair factors by binding to its catalytic domain. It not only kills ovarian cancer cells with DNA repair defects but also becomes sensitive to the cells to other DNA-damaging agents. Remarkably, COH34 is effective at killing ovarian cancer cells that are resistant to the PARP1 inhibitor.

#### 4.1.4. Esophageal Cancer

Sepantronium bromide (YM155) restrains esophageal squamous-cell carcinoma (ESCC) growth and keeps body weight in the ESCC xenografts mice model. The main reason is that YM155 activates PARP1, synthesizes PAR polymer, and stimulates AIF nucleus translocation. Genetic KO of PARP1 or AIF abolished YM155-induced parthanatos [132].

#### 4.1.5. Head and Neck Cancer (HNC)

Oral squamous cell carcinoma (OSCC), a kind of head and neck squamous cell carcinoma (HNSCC), is widespread in cancer [133,134,135]. Rucaparib, a PARP1 inhibitor, depolarizes the mitochondrial membrane potential, up-regulates PARP1, MIF, and AIF nucleus translocation, then attenuates parthanatos in OSCC cells. Furthermore, oxaliplatin suppresses OSCC cells by stimulating PARP1-mediated parthanatos via ascending ROS production [97].

#### 4.1.6. Glioma

It is reported that PARP1 status is positively correlated with the degree of glioma malignancy [136]. The cell death of the glioma, which is caused by H_2_O_2_, is accompanied by PAR polymer cytoplasmic formation, PARP1 over-activation, and AIF nuclear translocation. In addition, JNK activation facilitates glioma cell parthanatos through increasing intracellular ROS [137]. Glioma cell lines and mice model of xenograft glioma are used to investigate the parthanatos mechanism of deoxypodophyllotoxin (DPT) in cancer cell death, and the result reveals that DPT stimulates parthanatos in glioma cells through induction of overburdened ROS [98].

#### 4.1.7. Others

Meanwhile, dexmedetomidine suppresses bupivacaine-induced parthanatos in human SH-SY5Y cells via miR-7-5p/PARP1 axis-mediated ROS [99]. SH-SY5Y cell death and ropivacaine-induced apoptosis are related to parthanatos, ropivacaine induces NAD^+^ depletion and PARP1 activation, and the result of treatment with the PARP1 inhibitor PJ-34 indicates that NAD^+^ depletion is caused by PARP1 activation [100]. The hot water extract of Korean *ginseng* is reported to obviously inhibit MNNG-induced cell death, significantly reducing AIF expression and nucleation, and decreasing ROS levels in SH-SY5Y cells [138]. In the N-butyl-N-(4-hydroxybutyl)-nitrosamine (BBN) model, MIF accelerates bladder cancer by promoting cell proliferation and angiogenesis, so MIF inhibitors may be a helpful treatment in the field of this disease [139]. Hela cells’ death upon polybrominated diphenyl ethers quinone metabolite (PBDEQ) exposure results from increased ROS production, which manifests as PARP1 and AIF-mediated parthanatos [140].

### 4.2. Parthanatos and Retinal Disease

Hyper-activation of PARP enzymes is reported to participate in photoreceptor degeneration in the retinal detachment mouse model [101]. To explore the accurate association of PARP1 with photoreceptor cell death, researchers examined the phenotype of PARP1 KO retina in a mouse model. Their finding demonstrates that PARP1 is an indispensable part of normal retinal function and has essential significance for photoreceptor degeneration under pathological conditions. Furthermore, the result suggests that parthanatos is indispensable for retinal degeneration and highlights the importance of PARP1 inhibitors in treating retinitis pigmentosa (RP). The hyper-activation of PAR results in AIF release and then affects rod photoreceptor death—a certain genetic form of RP. In the RP mouse model, PAR up-regulation occurs only in the mutant retinas. RP mouse retinal explants, which are treated with a PARP inhibitor, show reduced cell stress and decreased responsiveness to markers of cell death [141]. Interestingly, researchers found proof in animals of some other retinal degeneration sustaining the role of parthanatos, but not other cell death [142]. NMNAT1-associated retinal degeneration (LCA9), a kind of retinal disease, is caused by mutations of the enzyme, which produces nuclear NAD^+^ [143]. Moreover, the NAD^+^ level is reduced, but the NAD^+^ precursor nicotinamide mononucleotide is augmented in the model, compared with littermate controls [144].

NAD^+^/ATP depletion is deemed to be a potential reason for dry age-related macular degeneration, and this premise is verified using a human-derived retinal pigment epithelium cell line (ARPE-19) that suffers from oxidative stress. PARP1 activity is increased in ARPE-19 cells after stimulation, but the expression of NAD^+^ and ATP is reduced, compared with the normal group. Mitochondrial dysfunction and organelle depolarization are changed by supplementing NAD^+^ or inhibiting PARP1 [145,146]. The photoreceptors are protected against visible-light-induced parthanatos by regulating the mTOR/PARP1 axis or the downstream factors of AIF, and mTOR interacts with PARP1 via sirtuin 1 to adjust parthanatos. On the one hand, the mTOR signaling pathway directly affects intracellular NAD^+^ levels, and on the other hand, it regulates NAD^+^ by regulating the expression and activity of SIRT1. In addition, the activity of SIRT1 is affected by the almost complete depletion of NAD^+^ and ATP pools caused by the over-activation of PARP1 [147]. Parthanatos occurs because of PARP1 over-activation and cellular NAD^+^ depletion, and the subsequent reduction in SIRT1 deacetylase activity, which is related to substrate deprivation and a high level of the reaction product nicotinamide. C2-acetylsphingosine induces photoreceptor death by parthanatos mechanisms, involving the activation of PARP1, the decline of mitochondrial membrane potential, and AIF translocation [148]. In response to oxidative stress, activated PARP1 consumes intracellular NAD^+^ and depletes ATP, resulting in the production of PAR polymers, the nucleus translocation of AIF, and lastly massive DNA fragmentation. PJ-34 is demonstrated to be a promising therapeutic agent that alleviates photoreceptor parthanatos death in retinal detachment by inhibiting the PARP1/AIF pathway [141].

### 4.3. Parthanatos and Neurological Diseases

Neurodegenerative diseases are typical of the enhancement of PARP1 expression and the accumulation of PAR. The activation of PARP1 results in the PAR-mediated AIF nucleus translocation. PAR accumulation and AIF nucleus translocation are associated with various neurological diseases. Alzheimer’s disease (AD) is featured with cognitive impairment, amyloid β (Aβ) production, and PARP1 activation. Abnormal Aβ leads to NO production on a large scale, resulting in PARP1 activation and DNA damage to sensitive subcellular structures [149]. PARP1 depletion protects the brain against Aβ-evoked microglia activation, hippocampal synaptic integrity alteration, and cognitive impairment using hAPPJ20 mice crossed with PARP1^−/−^ mice [150]. In an in vivo model, an inhibitor of PARP1 not only protected the brain against pro-oxidative processes [151] but also safeguarded against systemic inflammatory response-induced impairment of cognitive function, and observably improved spatial memory and locomotor activity in lipopolysaccharide-treated animals [152,153].

Parkinson’s disease (PD) is featured by dopaminergic neurons (DN). The latest study about PD proves that parthanatos is responsible for selective DN loss [154]. The neurons in PD patients show a marked nucleus translocation of AIF [155]. MPTP/1-methyl-4-phenylpyridinium (MPP^+^) involves PARP1 and AIF nucleus translocation [79,156,157]. The pharmacological inhibition of PARP1 reduces α-synuclein cytotoxicity and MPP^+^-induced cell death in the PD in vitro model [127,158,159]. In the MPTP animal model of PD, AIF implicates in mediating MPP^+^ toxicity in dopaminergic cells, which is prevented by the knockdown of AIF [160]. MPP^+^ causes dopaminergic neuronal cell death and increases protein arginine methyltransferases 1 (PRMT1) expression in a dopaminergic cell line. MPP^+^-induced cell death is preceded by PRMT1 knockdown, the expression and activity of PARP1, and arylation which are elevated by MPP^+^. Moreover, the expression of PRMT1 in the substantia nigra pars compacta of MPTP-injected mice is increased, and PRMT1 positively regulates nucleus translocation of AIF. In addition, MPTP-induced DN death is decreased in PRMT1 haploinsufficient (prmt1^+/^^−^) mice [161]. In human systems, PAR-mediated parthanatos is accountable for the drop of DN in PD [162]. In a word, PAR or PAR-AIF interaction targets will be a new promising measure for preventing DN loss in PD.

Parthanatos is involved in MCAO/R-induced model animals, which used different PARP inhibitors (PJ-34, olaparib, 3-AB, DPQ, INO-1001, FR247304, DR2313, MP-24, HYDAMTIQ) or PARP1-null mice (PARP1^−/−^) [163,164]. The oxygen-glucose deprivation (OGD)-injured mesenchymal stem cells (MSCs) neuron model is used to reveal that co-culturing with MSCs protects the cortical neurons from OGD-induced parthanatos by alleviating AIF nucleus translocation [7]. Remote limb preconditioning (RPC) has a protective effect on neuronal oxidative DNA damage and DNA fragmentation after ischemic stroke. RPC down-regulates the PAR/AIF pathway and neuronal parthanatos by suppressing PAR accumulation, AIF translocation, and AIF/H2AX interaction [165]. Astragaloside IV (AIV), a natural saponin abundant in *Astragalus membranaceus*, guards mitochondrial HK-II and reduces the release of AIF, resultantly protecting neurons from parthanatos in the MCAO model mouse [166].

### 4.4. Parthanatos and Diabetes

PARP1 hyper-activation is associated with many various characteristics of early nephropathy related to type 1 diabetes, which provides a theoretical basis for developing and studying PARP1 inhibitors and PARP1 inhibitor-containing combination therapies [102]. PARP1^−/−^ gene deficiency alleviates diabetic kidney disease by using the PARP1 deficient mouse to evaluate the role of PARP1 [167]. In the diabetic peripheral neuropathy (DPN) model, a hydrogen-rich medium effectively suppresses parthanatos by down-regulating the PAR level and AIF nucleus translocation [168]. Resveratrol (3,4′,5-trihydroxystilbene), an antioxidant and non-flavonoid polyphenolic compound, extracted from many natural sources, ameliorates type 1 diabetes mellitus (T1DM)-induced sperm abnormality and DNA damage [103].

### 4.5. Parthanatos and Renal Disease

Chemical inhibition of PARP1 activity reduces renal fibrosis [102]. PARP1 deficiency reduces cisplatin-induced kidney dysfunction, oxidative stress, pro-inflammatory gene induction, and parthanatos as well [169,170,171]. Renal I/R-induced injury or necrosis is decreased and renal function is preserved in mice, which are pretreated with PARP1 inhibitor or excised with the PARP1 gene [172,173,174,175]. PARP1 is involved in renal fibrosis by binding to the cellular communication network factor 2 promoter in mouse proximal tubule epithelial cells (formerly known as a connective tissue growth factor). Pretreatment with 3,3,5-triiodothyronine (T_3_) lowers the clinical and histological signs of renal I/R injury in rats and these signs are contacted with reductions in tubular PARP1 expression [104]. The PARP1 inhibitor 4-hydroxy quinazoline attenuates the disintegration of the tubulointerstitial structures in the acute kidney model [105]. PARP1 inactivation by treatment with PJ-34 contributes to the decrease in interstitial fibrosis in murine kidneys; therefore, PARP1 is different in obstructive and ischemic renal injury [106]. Using lupus and anti-glomerular basement membrane models of nephritis to determine the effect of PARP1 on renal inflammation, researchers found that the activation of PARP1 and subsequent necrotic cell death is involved in the pathogenesis of male glomerulonephritis [176]. PARP1 ablation preserves ATP levels and renal functions and attenuates inflammatory response in the setting of I/R injury in a mouse model [177].

Glyceraldehyde-3-phosphate dehydrogenase (GAPDH)-poly(ADP-ribosyl)ation suppresses glycolysis, exacerbates ATP depletion, and induces parthanatos in a model of renal I/R injury [178]. In addition to tubulotoxicity and I/R injury, acute tubule necrosis is partially prevented by different inhibitors of PARP1 in animals attacked with intravenous and intravascular lipopolysaccharide as a model of septic mediated renal failure [107,108,175].

### 4.6. Parthanatos and Cardiovascular Diseases

After myocardial I/R, ROS are produced and PARP1 is activated, while PARP1 deficiency improves cardiac contractility and recovers energy metabolites. PARP1 inhibitor alleviates the infarct size of rat heart and ameliorates morphology and function in myocardial I/R rats, for instance, mitigating myocardial necrosis and inflammation response [164].

Besides myocardial I/R, parthanatos promotes the progression of heart failure, PARP inhibitors effectually restore impaired myocardial function. L-2286 prevents cardiac remodeling, enhances systolic function, and delays the development of heart failure in a spontaneously hypertensive rat (SHR) model [109]. 3-aminobenzamide (3-AB) significantly protects against myocardial morphological and functional alterations in a rat model of myocardial infarction. It is worth noting that infarct size and circulating creatine kinase activity are decreased, and myocardial contractility is restored by 3-AB [110]. INO-1001 is confirmed to arrest the pressure overload-induced attenuation in cardiac contractility and reduce the collagen formation and AIF nuclear translocation in the model of transverse aortic constriction (banding)-induced heart failure [111]. Patients with chronic heart failure (CHF) and volunteers with a normal heart function are investigated for examining oxidative stress-related activation of PARP1. Markers of oxidative-nitrative stress, PARP1 activation, and AIF translocation in blood components are associated with reduced cardiac function and the clinical manifestations of CHF [179].

### 4.7. Parthanatos and Other Diseases

Many other diseases have a close connection with parthanatos. For instance, zVAD and Nec-1 treatment significantly augment the incidence of cleaved PARP in live shikonin-induced Jurkat cells and lessen the level of DNA damage, in which the characteristics of parthanatos appear in Jurkat cells [112]. APO866 is a small molecule drug that indirectly interferes with NAD^+^ biosynthesis. APO866 has anti-leukemia activity and induces a large number of ROS/RNS, which lies on PARP1 integrity. This provides a promising way to enhance the anti-tumor activity of APO866 by regulating the parthanatos pathway [113]. Another study points out that hepatocytes are indeed susceptible to parthanatos in vitro. PARP1 inhibitor restores impaired hepatocytes, which is caused by the induction of parthanatos. This provides an experimental basis for searching for potential therapeutic targets for acute and chronic liver diseases [180]. Researchers identified the involvement of parthanatos in remote lung injury due to I/R or acute immune rejection in the renal allografts [114]. Smoke-mediated activation of the parthanatos pathway increases in human bronchial epithelial cells originating from habitual smokers compared to non-smokers, and the use of a specific PARP1 inhibitor, BMN673, abolishes the effect of smoke-induced activation of the parthanatos [115]. In addition, parthanatos is related to age-related macular degeneration [145]. Furthermore, the link between parthanatos and inflammation aroused great attention. PARP1 is able to enhance the transcription of pro-inflammatory genes and down-regulate multiple simultaneous pathways of inflammation and tissue injury [14,45]. Therefore, some ultrapotent novel PARP inhibitors such as rucaparib, niraparib, and olaparib have entered human clinical trials.

## 5. Discussion and Perspectives

Parthanatos is regulated by PARP1 and activated by oxidative stress-induced DNA damage and chromatolysis. It should be strictly controlled at multiple indexes that include PARP1, NAD^+^, ATP, AIF, MIF, PAR, PARG, and ARH3. In a range of different disease models, several pharmacological agents or genetic manipulations are used to modulate parthanatic responses, often reducing morbidity and mortality. However, parthanatos is still in its infancy and there are some thorny issues in parthanatos research.

In many cases, investigators concluded that parthanatos participated in pathogenic processes due to PARP1, AIF, PARG, and ROS, resulting in the loss of cell membrane integrity and large DNA fragmentations. Although AIF, PARG, and ROS inhibitors have no toxic effects secure in preclinical animal studies, they are no clinical application till now. Related studies will help to guide better the development and application of AIF, PARG, and ROS inhibitors for different diseases. The activity of the PARP1 enzyme takes charge of PAR degradation and must be tightly controlled for avoiding either PAR accumulation or inappropriate PAR degradation. PARP1 is an integral part of the regulation of parthanatos: PARP1 activation will result in PAR polymer accumulation, AIF nucleus translocation, AIF-mediated chromatin condensation/DNA fragmentation, and parthanatos occurs. Autophagy has a hand in the occurrence of parthanatos. During autophagy, the activity of PARP1 is not affected and a large number of PARs are synthesized by PARP1, thus promoting ferroptosis [181]. Furthermore, PARP1 is involved in necroptosis, tumor necrosis factor (TNF)-α regulates necroptosis by inducing ATP depletion along with PARP1 activation. Ferroptosis and parthanatos are cell death caused by intracellular ROS overload and are associated with many diseases. Nowadays, inhibition of parthanatos is a pathway to avoid oxidative stress-induced cell death and is one of the strategies to intervene in the conditions of cardiovascular degeneration, diabetes, tumors, and so on. JNK, MAPK, and mTOR signaling pathways, which are activated by ROS, are vital to regulating cell parthanatos. Activated JNK, MAPK, and mTOR signaling pathways enhance the expression and the hyper-activation of PARP-1 via the improvement of intracellular ROS levels in cells. Hence, the regulation of these cell death modes has some similarities. However, their relationship remains an open question. Whether they can be integrated into a complete regulatory network is still to be further explored and studied. AIF nuclear translocation, a downstream event of PAR signaling, is also an attractive therapeutic target. They are critical steps of parthanatos and play pivotal roles in the marks therapy of cell death. However, what are the mechanical aspects of the AIF-releasing capacity of PAR? MIF possesses many vital advantages over PARP1 inhibition in some cases because long-term inhibition of PARP1 weakens the detection and restoration of DNA injury. Inhibiting MIF’s nuclease activity and interaction of MIF-AIF can bypass this potential problem and provide treatment opportunities for various diseases. However, how MIF interacts with AIF and what influences the interaction of MIF and AIF is still not clear.

Obviously, an in-depth study of the parthanatos pathway is of great significance to explore the pathogenesis and prevention of diseases. The research of drugs that target parthanatos will become a hot spot in the clinical medicine and pharmaceutical industry. Recently, the progress of parthanatos has been greatly achieved in cancer, retinal disease, diabetes, renal disease, heart failure, myocardial infarction, leukemia, lung injury, smoke-related lung diseases, stroke, ischemic tissue injury, brain trauma, and neurodegenerative diseases. For instance, PARP1 inhibitors (olaparib, rucaparib, niraparib, and talazoparib) are approved by the Food and Drug Administration (FDA) for clinical use in ovarian or breast cancer, and the variety of treated tumors is growing. interestingly, PARP1 agonists such as β-lapachone and deoxypodophyllotoxin are reported to trigger parthanatos in hepatoma carcinoma cells and glioma cells through indirectly activating PARP1 via induction of excessive ROS. These compounds, as well as PDD00017273 (acting on PARG) and 3-AB (acting on PARP1), show great potential and prospect for clinical application. To sum up, the discovery of parthanatos carves a new plat of disease research.

## Figures and Tables

**Table 1:** Similarities and differences between apoptosis, necrosis, autophagy, and parthanatos.

Features	Parthanatos	Apoptosis	Necroptosis	Autophagy
Morphological features	Dissipation of the inner transmembrane potential, nuclear and chromatin condensation	Cellular and nuclear volume reduction, chromatin agglutination, nuclear fragmentation, formation of apoptotic bodies and cytoskeletal disintegration, no significant changes in mitochondrial structure	Plasma membrane breakdown, generalized swelling of the cytoplasm and organelles, moderate chromatin condensation, spillage of cellular constituents into the microenvironment	Formation of double-membraned autolysosomes, including macroautophagy, microautophagy and chaperone-mediated autophagy
Biochemical features	DNA injury, energy depletion and PAR accumulation	DNA fragmentation	Drop in ATP levels	Increased lysosomal activity
Regulatory pathways	PARP1/AIF signaling pathway	Death receptor pathway, mitochondrion pathway and endoplasmic reticulum pathway; caspase, P53, Bcl-2-mediated signaling pathway	Tumor necrosis factor type 1 (TNF-R1) and Receptor-interacting protein 1 (RIP1)/RIP3-mixed-lineage kinase domain-like (MLKL) related signaling pathways; protein kinase C (PKC)-mitogen-activated protein kinase (MAPK)-activatorprotein1 (AP1) related signaling pathway; ROS-related metabolic regulation pathway	Molecular target of rapamycin (mTOR), Beclin-1, P53 signaling pathway
Key genetic inhibition or inhibition by protein overexpression	PARP1 knockout, AIF down-regulation (e.g., in Harlequin mouse)	Bcl-2 overexpression, Inhibition of caspases (3, 8, and 9), Inhibition of PP2Ad, CrmA expression	Inhibition of RIP1 or RIP3	Inhibition of Activating molecule in BECN1-regulated autophagy protein 1 (AMBRA1), Recombinant human autophagy related 5/7/12 (ATG5/7/12), or Recombinant Beclin 1 (BECN1)
Examples of trigger factors and/or conditions	Excitotoxicity Ischemia Stroke Reactive oxygen/nitrogen species	Death receptor signaling Dependence receptor signaling DNA damage Trophic factor withdrawal Viral infections	Excitotoxicity Ischemia Stroke Reactive oxygen/nitrogen species	Amino acid starvation Serum starvation Protein aggregates

**Table 2:** Summary of parthanatos in animal/human models of human pathological conditions of related diseases.

Related Diseases	Evidence of Parthanatos Involvement in Disease	Models	Inhibitors	Outcome of PARP1 or Parthanatos Pathway Inhibition in Animal Models/Observation	Ref.
Breast cancer	PARP1	Patients with advanced breast cancer	Talazoparib, BZL101	Improvements and significant delays in the time to clinically meaningful deterioration according to both the global health status-quality-of-life and breast symptoms scales were observed.	[85,86]
	AIF	Patients with metastatic breast cancer	Ganetespib	Shows evidence of activity in metastatic HER2-positive and triple-negative breast cancer.	[87]
	AIF	ErbB2 transgenic mouse FVBN-Tg; SK-BR-3 cells, MDA-MB-231 cells	GA/17AAG and Lapatinib	Shows evidence of activity in metastatic HER2-positive and triple-negative breast cancer.	[88,89]
Colon cancer	PARP1	PARP1^−/−^ and PARP1^+/+^ cells (A549, LoVo, and SW620) and mice	AG14361	Increases the antiproliferative activity, inhibits recovery from potentially lethal γ-radiation damage.	[90]
	PARP1	SW613-B3 colon carcinoma cells	5-(N,N-hexamethylene amiloride) (HMA)	AIF nuclear translocation.	[91]
Ovarian cancer	PARP1	Patients with platinum-sensitive, relapsed serous ovarian cancer	Olaparib, niraparib and rucaparib	Prolong median duration of progression-free survival.	[92,93,94]
	PARG	Kuramochi, OVSAHO, COV362, COV318, CAOV3, and OVCAR3 cell lines	PDD00017273	Induces increased DNA damage in cancer cells.	[95]
	PARP1	SF9 cells	COH34	Binds to the catalytic domain of PARG, thereby prolonging PARylation at DNA lesions and trapping DNA repair factors.	[96]
Oral squamous cell carcinoma	PARP1	CAL27 and SCC25 cells; Athymic nude mice	Oxaliplatin	Inhibits the proliferation and migration of OSCC cells in vitro, and also inhibits the tumorigenesis in vivo.	[97]
Melanoma	ROS	Rat C6, and human SHG-44 and U87 glioma cells; SH-SY5Y cells	Deoxypodophyllotoxin; dexmedetomidine; Korean ginseng	Induces glioma cell death and inhibits the growth of xenograft glioma; counteracted bupivacaine-induced changes of mitochondrial membrane potential and ROS production.	[98,99]
	PARP1	SH-SY5Y cells	PJ-34	Inhibits intracellular NAD^+^ depletion.	[100]
Retinal disease	PARP1	Retinal disease rats	PJ-34	The structure and outer nuclear layer (ONL) thickness of retinas are preserved, and the photoreceptors death is decreased.	[101]
Diabetes	PARP1	Streptozotocin-diabetic rats	1,5-isoquinolinediol (ISO), 10-(4-Methyl-piperazin-1-ylmethyl)-2H-7-oxa-1,2-diaza-benzo[de] anthracen-3-one (GPI-15427)	Prevents the increase in urinary albumin excretion.	[102]
	PARP1	Streptozotocin-induced rat testes	Trans-resveratrol	Mitigates type 1 diabetes mellitus-induced sperm abnormality and DNA damage.	[103]
Renal disease	PARP1	I/R-injured rats	3,3,5 triiodothyronine (T3)	Improves acute tubular necrosis.	[104]
	PARP1	Acute kidney rejection rats	4-hydroxy_x0002_quinazoline (4OHQ)	Protects tubulointerstitial region.	[105]
	PARP1	I/R-induced mouse kidneys	PJ-34	Reduces ischemic acute kidney injury and interstitial fibrosis.	[106]
	PARP1	LPS-induced mice	Olaparib	Restores serum levels of urea, creatinine, and uric acid to normal.	[107]
	PARP1	Endotoxic shock-induced canine	3-aminobenzamide (3-AB)	Improves systemic hemodynamics, renal hemodynamics, renal oxygen metabolism, and renal tubular cell apoptosis.	[108]
heart failure	PARP1	Spontaneously Hypertensive rat model of heart failure	L-2286	Improves gravimetric parameters, cardiac fibrosis, and several echocardiographic parameters and delay the onset of hypertension-induced HF without lowering blood pressure.	[109]
	PARP1 and AIF	Transverse aortic constriction (banding)-induced mice	INO-1001	Prevents the pressure overload-induced decrease in cardiac contractile function, attenuate the formation of collagen in the hearts.	[110]
Myocardial infarction	PARP1	Myocardial I/R-injured rats	3-AB	Reduces infarct size, attenuates circulating creatine kinase activity, and restores myocardial contractility.	[111]
Leukemia	PARP1	Jurkat cells	Necrostatin-1 (Nec-1)	Increases incidence of cleaved PARP and reduces levels of DNA damage.	[112]
	ROS/RNS	Jurkat, Molt-4, ML-2 and THP-1 cells	APO866	Contributes substantially to the antileukemia effect.	[113]
Lung injury	PARP1	Human proximal tubular HK-2 cells and human lung alveolar epithelial A549 cells; renal I/R rats	Necrostatin-1 (nec-1) or/and 3-AB	Improve lung injury.	[114]
Smoke-related lung diseases	PARP1	Human bronchial epithelial (HBE) cells	BMN673	Inhibits translocation of AIF and EndoG to the nucleus.	[115]
Stroke	Poly(ADP-ribosyl)ation	Middle cerebral artery occlusion (MCAO)-induced rats	INO-1001	Reduces infarct size and improves neurological status.	[116]
	PARP1	MCAO-induced Sv129 mice	PJ-34	Reduces infarct size, improves neurological status.	[117]
	PARP1	MCAO-induced rats	3-AB	Reduces infarct volume	[118]
	PARP1	MCAO-induced rats	3-AB	Reduction in NMDA-induced glutamate elevation.	[119]
	Poly(ADP-ribosyl)ation	Global cerebral ischemia rats	PJ34	Inhibition of microglia/macrophage activation, decrease in CA1 neuronal death after forebrain ischemia.	[120]
Ischemic tissue injury	Poly(ADP-ribosyl)ation	MCAO-induced Sv129 rats	3,4-dihydro 5-[4-(l-piperidinyl) butoxy] I (2H)-isoquinolinone	Reduces infarct size.	[121]
	Poly(ADP-ribosyl)ation	MCAO-induced rats	3-AB	Decreases infarction volume.	[122]
	PARP1	MCAO-induced rats	3-AB	Decreases infarction volume.	[123]
	PARP1	MCAO-induced rats	Cilostazol	Reduction in infarct size, nuclear AIF translocation and apoptosis after MCAO followed by reperfusion.	[124]
	PARP1	MCAO-induced mice	3-AB	Neuroprotection, decrease in infarct volume, improvement of neurological score.	[125]
	PARP1	MCAO-induced mice	3-AB	Decreases infarction volume.	[71]
Brain trauma	PARP1	Global cerebral ischemia gerbils	3-AB	Robust neuroprotection in CA1 neurons after 3 min ischemia, reduces forebrain ischemia.	[126]
Neurodegenerative diseases	PARP1	MPTP-induced C57B1/6 mice	Benzamide	Reduces neuronal death.	[127]
Peripheral nerve injury	Poly(ADP-ribosyl)ation	Chronic constriction injury SD rats	Benzamide	Reduces neuropathic pain.	[128]

## Data Availability

Not applicable.
